# Obesity in adults: overview of a clinical practice guideline for South Africa

**DOI:** 10.3389/fendo.2026.1875018

**Published:** 2026-10-12

**Authors:** Marli Conradie-Smit, Wayne May, Clayton Arendse, Jason Bantjes, Zaheer Bayat, Elna Barnard, Sindeep Bhana, Nomathemba Chandiwana, Annibale Cois, Paula N. Diab, Zodwa Dire, Virginia R. Fourie, Julia H. Goedecke, Jocelyn Hellig, Zachary Koto, Kathleen Mawson, Makama Andries Monyeki, Angela Murphy, Christina Nieuwoudt, Marisa Noeth, Nicole Pearce, Joanna Skelton, Thinus (JGM) Smit, Sanjeev Sockalingam, Francois H. van Zyl, Jeanne Lubbe

**Affiliations:** 1 Division of Endocrinology, Faculty of Medicine and Health Sciences, Stellenbosch University and Tygerberg Academic Hospital, Cape Town, South Africa; 2 Cape Town Bariatric Clinic, Kingsbury Hospital, Cape Town, South Africa; 3 Department of Psychiatry, Stellenbosch University and Department of Psychology Tygerberg Academic Hospital, Cape Town, South Africa; 4 Mental health, Alcohol, Substance use and Tobacco Research Unit, South African Medical Research Council; Department of Psychiatry and Mental Health, University of Cape Town; Institute for Life Course Health Research, Department of Global Health, Stellenbosch University, Cape Town, South Africa; 5 Division of Endocrinology, Department of Internal Medicine, Helen Joseph Tertiary Hospital and University of the Witwatersrand, Johannesburg, South Africa; 6 M-Care Optima Bloemcare Psychiatric Hospital, Bloemfontein, South Africa; 7 Center for Advance Medicine, Johannesburg; Chris Hani Baragwanath Academic Hospital, University of Witwatersrand, Johannesburg, South Africa; 8 Desmond Tutu HIV Centre, University of Cape Town, Cape Town, South Africa; 9 Burden of Disease Research Unit, South African Medical Research Council; Division of Epidemiology and Biostatistics, School of Public Health, University of Cape Town, Cape Town, South Africa; 10 Atrium Diabetes Center, Gillits, Kwazulu-Natal; Dept of Family Medicine University of Pretoria, Pretoria, South Africa; 11 Division of Endocrinology, Department of Internal Medicine, Chris Hani Baragwanath Academic Hospital and University of the Witwatersrand, Johannesburg, South Africa; 12 Biomedical Research and Innovation Platform, South African Medical Research Council, Cape Town, South Africa; 13 Life Kingsbury Hospital, Cape Town, South Africa; 14 Department of Surgery, Sefako Makgatho Health Science University and Dr George Mukhari Academic Hospital, Ga-Rankuwa, Pretoria, South Africa; 15 Department of Psychiatry, Stellenbosch University and Tygerberg Academic Hospital, Cape Town, South Africa; 16 Physical Activity, Sport and Recreation (PhASRec), Faculty of Health Sciences, North-West University, Potchefstroom, South Africa; 17 Sunward Park Medical Center, Boksburg, South Africa; 18 Zuid Afrikaans Hospital, Pretoria, South Africa; 19 Zuid-Afrikaans Hospital; Dept Internal Medicine University of Pretoria, Pretoria, South Africa; 20 Obesity Canada, Edmonton, AB, Canada; 21 Busamed Gateway Private Hospital, Umhlanga, Durban, South Africa; 22 Zuid Afrikaans Hospital, Obesity Health Centre; Department of Surgery, Sefako Makgatho Health Sciences University, Pretoria, South Africa; 23 Department of Psychiatry, University of Toronto, Centre for Addiction and Mental Health, Toronto, ON, Canada; 24 Bariatric Surgery Program, University Health Network, Toronto, ON, Canada; 25 Private Practice and CPR Trial Centre; Mediclinic Private Hospital, Worcester, South Africa; 26 Division Surgery, Department of Surgical Sciences, Stellenbosch University and Tygerberg Academic Hospital, Cape Town, South Africa

**Keywords:** adult, Africa, obesity, South Africa, treatment

## Abstract

Obesity is a multifactorial condition posing an escalating public health burden in South Africa, where access to effective, evidence-based interventions remains constrained. This overview describes the South African adaptation of the Canadian Adult Obesity Clinical Practice Guideline, contextualized through locoregional evidence to provide pragmatic, setting-appropriate recommendations and culminating in a comprehensive 18-chapter guideline. Key areas of uncertainty — including HIV-associated weight trajectories and culturally specific perspectives on obesity — are acknowledged, with guidance offered where evidence permits. This guideline addresses a critical gap in culturally responsive, resource-sensitive obesity management within the African healthcare context.

## Introduction

Obesity is driven by complex factors and represents a growing public health challenge especially in South Africa. Despite rising prevalence, evidence-based treatment guidelines remain scarce, and access to effective interventions is limited in resource constrained settings (1, 2). This overview reports on the South African adaptation of the Canadian Adult Obesity Clinical Practice Guideline (CPG). Drawing on locoregional evidence, we offer pragmatic, contextually interpreted guidance for South Africa, highlighting local research gaps and controversies. Where evidence is limited — particularly around HIV-associated weight trajectories and cultural perspectives — we acknowledge uncertainties while providing feasible recommendations. This guideline addresses the need for culturally appropriate, resource-sensitive obesity management in an African healthcare setting.

### Guideline development

The South African Clinical Practice Guideline for Treatment of Adult Obesity was developed by a multidisciplinary committee (endocrinologists, surgeons, public-health practitioners, mental health experts, dietitians, primary-care clinicians, medical researchers and individuals with lived experience) and followed a pre-specified process (3, 4). Professional and patient society invitations and endorsements were secured, and active engagement with government established. Ethical approval was granted by Stellenbosch University’s Health Research Ethics Committee (Ref. X24/07/020).

A hybrid GRADE-ADAPTE approach was used: GRADE principles guided appraisal of evidence quality and recommendation strength; the ADAPTE framework provided structured contextualization without *de novo* development when suitable guidance existed in the Canadian guideline (5). Decision rules were standardized to (1): adopt fully applicable recommendations (2); adapt recommendations requiring contextual changes; and (3) exclude recommendations with regulatory, availability, or feasibility barriers. Where updated external consensus statements altered the evidence landscape, such as for indications for metabolic and bariatric surgery (MBS), recommendations were revised with permission.

Internal peer review ensured consistency of chronic disease framing and person-first language, clarity of clinician actions in resource-variable (public vs private) settings, and alignment with South Africa’s Obesity and Non-Communicable Disease (NCD) strategies (6). External peer-review was performed by Obesity Canada with further chapter-specific blinded peer review performed by local reviewers. The guideline was published in a special issue of the *South African Medical Journal* in November 2025 (7, 8). This article serves as an overview of the published guideline, specifically highlighting aspects for future review.

Dissemination is planned by means of professional training workshops and a planned postgraduate qualification, integration into medical curricula, partnerships with primary-care networks and healthcare facilities, and policy engagement with provincial and national health departments and funders. Decision-support tools, care pathways, multilingual patient resources, and audit indicators are in development with societal and digital experts— especially pertinent given the rapidly evolving pharmacotherapy landscape.

### Guideline chapters

#### Reducing weight bias in obesity management, practice and policy

Weight bias, stigma, and discrimination are pervasive, harming people living with obesity (PLWO) across society and healthcare (9). Explicit, implicit, and internalized bias are linked to care avoidance, poorer mental health, maladaptive coping, adverse physiological markers, and higher mortality (10). Clinician bias reduces access to appropriate care, patient education, and preventive screening, while narratives oversimplifying obesity as individual choice reinforce stereotypes and widen inequities (10, 11).

Effective bias-reduction requires multifaceted strategies: education on non-modifiable causes, empathy-building, peer-modeling, empowerment, and clinic environmental changes (9, 12).

Policymakers should avoid stigmatizing messaging, reject weight as a behavior-change target, and support anti-discrimination measures.

Recommendations were adopted from the Canadian CPG (**Table 1**).

**Table 1:** Recommendations and contextualisation.

Chapter	Recommendations	Local Context Key Points
Reducing weight bias in obesity management, practice and policy	HCPs should assess their own attitudes and beliefs regarding obesity and consider how their attitudes and beliefs may influence care delivery. (Level 1a; Grade A) HCPs should recognise that internalised weight bias (bias towards oneself) in PLWO can affect behavioural and health outcomes. (Level 2a; Grade B) HCPs should avoid using judgemental words, images and practices when working with PLWO. (Level 1a (words); Level 2b (images); Level 2a (practices); Grade A (words); Grade B (images/practices) We recommend that HCPs avoid making assumptions that an ailment or complaint a patient presents is related to their body weight. (Level 3; Grade C)	Pragmatic actions to reduce weight bias in clinical settings: Person−first language Staff training Physically accessible environments Appropriate equipment Patient advocacy
Epidemiology of adult obesity	HCPs should recognise and treat obesity as a chronic disease, caused by abnormal or excess body fat accumulation (adiposity) that impairs health, with increased risk of premature morbidity and mortality. (Level 2b; Grade B) The development of evidence-informed strategies at the health system and policy level should be directed at managing obesity in adults. (Level 2b; Grade B) Continued longitudinal national and regional surveillance of obesity that includes self-reported and measured data (i.e. height, weight, waist circumference) should be conducted on a regular basis. (Level 2b; Grade B)	Obesity prevalence in SA rose by 38% Women 29.5%%→42.6% Men 10.1% →12.1% Obesity in SA accounted for: 11.1% of total deaths 6.9% of disability The cost of obesity in SA: Public Sector ZAR33.194 million in 2020 Private Sector ZAR21.8 billion in 2022 National data is essential, yet large epidemiological data gaps exist in SA
Enabling participation in activities of daily living for people living with obesity	We recommend that HCPs ask PLWO if they have concerns about managing self-care activities such as bathing, getting dressed, bowel and/or bladder management, skin and/or wound care, foot care. (Level3; Grade C) We recommend that HCPs assess falls risk in PLWO, as this could interfere with their ability to participate in physical activity and their interest in doing so. (Level 3; Grade C)	Screen for fall risk by means of the Falls Efficacy Scale (13) Perform skin integrity screening in skin folds Aim to establish referral pathways to OT/Physio/Biokinetics
Assessment of people living with obesity	We suggest that HCPs involved in screening, assessing and managing PLWO use the ‘5As’ framework to initiate the discussion by asking for their permission and assessing their readiness to initiate treatment. (Level 4; Grade D) HCPs can measure height and weight and calculate BMI in all adults (Level 2a; Grade B), and measure waist circumference in individuals with a BMI 25 - 35 kg/m2. (Level 2a; Grade B (BMI); Level 2b; Grade B (waist circumference)) We suggest that a comprehensive history to identify root causes of weight gain as well as complications of obesity and potential barriers to treatment be included in the assessment. (Level 4; Grade D) We recommend measurement of blood pressure in both arms, fasting glucose or glycated haemoglobin and lipid profile to determine cardiometabolic risk and, where appropriate, alanine transaminase to screen for metabolic dysfunction-associated steatotic liver disease in PLWO. (Level 3; Grade D) We suggest that HCPs consider using the Edmonton Obesity Staging System to determine the severity of obesity and guide clinical decision-making. (Level 4; Grade D)	Initiate discussion with the 5As Framework (14) Combine BMI measurement with one of the following: Waist circumference Waist-to-height ratio Use the STOP-BANG score to screen for OSA (15) Use the FIB-4 Score to screen for steatotic liver disease (16) Make use of a staging tool: Edmonton Obesity Staging System (17) 4Ms Red flags to prompt specialist care referral: BMI>40kg/m^2^ Presence of T2D Threatened mobility
The role of mental health in obesity management	We recommend regular monitoring of weight, glucose and lipid profile in people with a mental health diagnosis who are taking medications associated with weight gain. (Level 3; Grade C) Healthcare providers can consider both efficacy and effects on body weight when choosing psychiatric medications. (Level 2a; Grade B) Metformin and psychological treatment, such as CBT, should be considered for prevention of weight gain in people with severe mental illness who are treated with antipsychotic medications associated with weight gain. (Level 1a; Grade A)	Screen for the presence of: Depression Anxiety Binge Eating Disorder ADHD Be aware of antipsychotic-induced weight gain. Evidence gaps: Guidance on metabolically informed prescribing in mental illness and locally informed interventions for antipsychotic-induced weight gain.
Medical nutrition therapy (MNT) in obesity management	We suggest that nutrition recommendations for adults of all body sizes should be personalised to meet individual values, preferences and treatment goals to support a dietary approach that is safe, effective, nutritionally adequate, culturally acceptable and affordable for long-term adherence. (Level 4; Grade D) PLWO should receive individualised MNT provided by a registered dietitian (when available) to improve weight outcomes (body weight, BMI), waist circumference and glycaemic control, and to establish lipid and BP targets. (Level 1a; Grade A) PLWO and impaired glucose tolerance (prediabetes) or T2DM may receive MNT provided by a registered dietitian (when available) to reduce body weight and waist circumference and improve glycaemic control and BP. (Level 2a; Grade B) PLWO can consider any of the many medical nutrition therapies to improve health-related outcomes, choosing the dietary patterns and food-based approaches that support their best long-term adherence. (Level 2-3; Grade B-C) PLWO and impaired glucose tolerance (prediabetes) should consider intensive behavioural interventions that target 5 - 7% weight loss to improve glycaemic control, BP and blood lipid targets, reduce the incidence of T2DM and microvascular complications (retinopathy, nephropathy and neuropathy) and reduce cardiovascular and all-cause mortality. (Level 1a (BP and lipids); Level 1a (T2DM); Level 1a (microvascular complications); Grade A (BP and lipids); Grade A (T2DM); Grade B (microvascular complications)) PLWO and T2DM should consider intensive behavioural therapy that targets 7 - 15% weight loss to increase the remission of T2DM and reduce the incidence of nephropathy, OSA and depression. (Level 1a (T2DM); Level 1a (nephropathy); Level 1a (OSA); Level 1a (depression); Grade A (T2DM); Grade A (nephropathy); Grade A (OSA); Grade A (depression)) We recommend a non-restrictive dietary approach to improve QoL, psychological outcomes (general wellbeing, body image perceptions), cardiovascular outcomes, body weight, physical activity, cognitive restraint and eating behaviours. (Level 3; Grade C)	MNT focuses on improving and maintaining health rather than solely achieving weight loss There is no single best dietary approach Success depends on long-term adherence (sustainability)
Physical activity in obesity management	Aerobic PA (30 - 60 minutes of moderate to vigorous intensity most days of the week) can be considered for adults who want to: achieve small amounts of body weight and fat loss achieve reductions in abdominal visceral fat and ectopic fat such as liver and heart fat, even in the absence of weight loss favour weight maintenance after weight loss favour the maintenance of fat-free mass during weight loss increase cardiorespiratory fitness and mobility (Level 2a (weight); Level 1a (visceral fat); Level 1a (ectopic fat); Level 2a (weight maintenance); Level 2a (fat-free mass maintenance); Level 2a (fitness); Level 2a (mobility); Grade B (weight); Grade A (visceral fat); Grade A (ectopic fat); Grade B (weight maintenance); Grade B (fat-free mass maintenance); Grade B (fitness); Grade B (mobility)) For adults living with overweight or obesity, resistance training may promote weight maintenance or modest increases in muscle mass or fat-free mass and mobility. (Level 2a; Grade B) Increasing exercise intensity, including high-intensity interval training, can achieve greater increases in cardiorespiratory fitness compared with moderate-intensity aerobic activity, and reduce the amount of time required to achieve similar benefits. (Level 2a; Grade B) Regular PA, with and without weight loss, can improve many cardiometabolic risk factors in adults who have overweight or obesity, including: hyperglycaemia and insulin sensitivity high BP dyslipidaemia (Level 2b (hyperglycaemia); Level 1a (BP); Level 2a (dyslipidaemia); Grade B (hyperglycaemia); Grade B (BP); Grade B (dyslipidaemia)) Regular PA can improve health-related quality of life, mood disorders (i.e. depression, anxiety) and body image in adults with overweight or obesity. (Level 2b; Grade B)	Recommended strategies: 150 –300 min of moderate-to-vigorous aerobic activity weekly Resistance training twice weekly Minimizing sedentary time Physical activity indicated for health benefit, as opposed to weight loss
Effective psychological and behavioural interventions in obesity management	Multi-component psychological interventions (combining behaviour modification [goal setting, self-monitoring, problem solving], cognitive therapy [reframing] and values-based strategies to alter nutrition and activity) should be incorporated into care plans for weight loss and improved health status and QoL in a manner that promotes adherence, confidence and intrinsic motivation. (Level 1a; Grade A) HCPs should provide longitudinal care with consistent messaging to PLWO to support the development of confidence in overcoming barriers (self-efficacy) and intrinsic motivation (personal, meaningful reasons to change), to encourage the patient to set and sequence health goals that are realistic and achievable to self-monitor behaviour and to analyse setbacks using problem solving and adaptive thinking (cognitive reframing), including clarifying and reflecting on values-based behaviours. (Level 1a (goals); Level 1a (self-monitor); Level 1a (setback analysis); Grade A (goals); Grade A (self-monitor); Grade A (setback analysis)) HCPs should ask patients’ permission to educate them that success in obesity management is related to improved health, function and QoL resulting from achievable behavioural goals, and not the amount of weight loss. (Level 1a; Grade A) HCPs should provide follow-up sessions consistent with repetition and relevance to support the development of self-efficacy and intrinsic motivation. Once an agreement to pursue a behavioural path has been established (health behaviour and/or medication and/or surgical pathways), follow-up sessions should repeat the above messages in a fashion consistent with repetition (the provider role) and relevance (the patient role) to support the development of self-efficacy and intrinsic motivation. (Level 1a; Grade A)	Effective techniques: Self-monitoring (food/activity diaries, self-weighing) Stimulus control Behavioural reinforcement Motivational Interviewing Cognitive Behavioral Therapy
Pharmacotherapy for obesity management	Pharmacotherapy for obesity management can be used for individuals with a BMI ≥30 kg/m2, or ≥27 kg/m2 with adiposity-related complications, in conjunction with medical nutrition therapy, physical activity and psychological interventions (semaglutide 2.4 mg weekly, liraglutide 3.0 mg daily, naltrexone/bupropion 16 mg/180 mg twice a day [BID], orlistat 120 mg three times a day [TID]). (Level 1a (semaglutide); Level 2a (liraglutide); Level 2a (naltrexone/bupropion); Level 2a (orlistat); Grade A (semaglutide); Grade B (liraglutide); Grade B (naltrexone/bupropion); Grade B (orlistat)) Pharmacotherapy may be used to maintain weight loss and to prevent weight regain (liraglutide 3.0 mg daily, orlistat 120 mg TID). (Level 2a (liraglutide); Level 2a (orlistat); Grade b (liraglutide); Grade B (orlistat)) Pharmacotherapy for obesity management in conjunction with health behaviour changes for people living with prediabetes and overweight or obesity (BMI ≥27 kg/m2) can be used to delay or prevent T2DM (liraglutide 3.0 mg daily, orlistat 120 mg TID). (Level 2a (liraglutide); Level 2a (orlistat); Grade B (liraglutide); Grade B (orlistat)) Obesity pharmacotherapy can be used in conjunction with health behaviour changes in people living with T2DM and a BMI ≥27 kg/m2, for weight loss and improvement in glycaemic control (semaglutide 2.4 mg weekly, liraglutide 3.0 mg daily, naltrexone/bupropion 16 mg/180 mg BID, orlistat 120 mg TID). (Level 1a (semaglutide); Level 1b (liraglutide); Level 2a (latrexone/bupropion); Level 2a (orlistat); Grade A (semaglutide); Grade A (liraglutide); Grade B (naltrexone/bupropion); Grade B (orlistat)) Pharmacotherapy can be considered in conjunction with health behaviour changes in treating people with OSA and a BMI ≥30 kg/m2, for weight loss and associated improvement in the apnoea-hypopnoea index (liraglutide 3.0 mg daily). (Level 2a; Grade B) Pharmacotherapy can be considered in conjunction with health behaviour changes in treating people living with MASLD and overweight or obesity, or for weight loss and improvement of liver parameters (liraglutide 1.8 mg daily, semaglutide 2.4 mg). (Level 3 (liraglutide); Level 4 (semaglutide); Grade C (liraglutide); Grade D (semaglutide)) Metformin and psychological treatment should be considered for prevention of weight gain in people with severe mental illness who are treated with antipsychotic medications associated with weight gain. (Level 1a; Grade A) For people living with overweight or obesity who require pharmacotherapy for other health conditions, we suggest choosing medications that are not associated with weight gain. (Level 4; Grade D) We do not suggest the use of prescription or over-the-counter medications other than those approved in SA for obesity management. (Level 4; Grade D)	Expected weight loss differs per agent (**Table 2**) Weight regain after discontinuation Continued updated review and recommendations as new agents become available
Metabolic and bariatric surgery: Selection and preoperative work-up	We suggest that a comprehensive medical and nutritional evaluation be completed and nutrient deficiencies corrected in candidates for MBS. (Level 4; Grade D) Pre-operative smoking cessation can minimize post-operative complications (Level 2a; Grade B) We suggest screening for and treatment of obstructive sleep apnoea in people seeking MBS. (Level 4; Grade D) Preoperative smoking cessation can minimise postoperative complications. (Level 2a; Grade B)	Most robust long-term treatment option (18) Indications (19): BMI >35 kg/m² regardless of comorbidities BMI 30–34.9 kg/m² with metabolic disease
Metabolic and bariatric surgery: Surgical options and outcomes	MBS is recommended for individuals with a BMI >35 kg/m2, regardless of the presence, absence or severity of comorbidities to: reduce long-term overall mortality induce significantly better long-term weight loss compared with medical management alone induce control and remission of T2DM, in combination with best medical management, over best medical management alone significantly improve QoL induce long-term remission of most obesity-related diseases, including dyslipidaemia, BP and MASLD. (Level 2b (mortality); Level 1a (weight loss); Level 2a (T2DM); Level 3 (QoL); Level 3 (dyslipidaemia); Level 3 (BP); Level 3 (MASLD); Grade B (mortality); Grade A (weight loss); Grade B (T2DM); Grade C (QoL); Grade C (dyslipidaemia); Grade C (BP); Grade C (MASLD)) MBS should be considered for individuals with metabolic disease and a BMI of 30 - 34.9 kg/m2. (Level 2a; Grade B) We suggest that the choice of metabolic and bariatric procedure be decided according to the patient’s need, in collaboration with an experienced MDT. (Level 4; Grade C) We suggest that adjustable gastric banding should not be offered owing to unacceptable complications and long-term failure. (Level 4; Grade D) We suggest that one-anastomosis gastric bypass should not be routinely offered owing to long-term complications in comparison with standard Roux-en-Y gastric bypass. (level 4; Grade D)	Total weight loss of 20%-40% Best evidence for improved cardiovascular outcomes and decreased mortality (20) Options: Sleeve Gastrectomy Roux-en-Y gastric bypass Endoscopic Sleeve Gastroplasty One Anastomosis Gastric Bypass Single-anastomosis duodenal-ileal bypass with sleeve gastrectomy
Metabolic and bariatric surgery: Postoperative management	HCPs can encourage PLWO who have undergone MBS to participate in and maximise their access to behavioural interventions and allied health services at an MBS centre. (Level 2a; Grade B) We suggest that MBS centres communicate a comprehensive care plan to primary HCPs for patients who are discharged, including MBS procedure, emergency contact numbers, annual blood tests required, long-term vitamin and mineral supplements, medications, behavioural interventions, and when to refer back. (Level 4; Grade D) We suggest that after a PLWO has been discharged from the MBS centre, HCPs should annually review nutritional intake, activity, compliance with multivitamin and mineral supplements and weight, as well as assess comorbidities, order laboratory tests to assess for nutritional deficiencies, and investigate abnormal results and treat as required. (Level 4; Grade D) We suggest that HCPs consider referral back to the MBS centre or to a local bariatric medicine specialist for technical or gastrointestinal symptoms, nutritional issues, pregnancy, psychological support, weight regain, or other medical issues related to MBS as described in this chapter. (Level 4; Grade D) We suggest that MBS centres provide appropriate follow-up and laboratory tests at regular intervals after surgery with access to appropriate HCPs (dietitian, nurse, social worker, surgeon, bariatric physician, psychologist/psychiatrist) until discharge/referral to primary care level is deemed appropriate for the patient. (Level 4; Grade D)	Annual micronutrient monitoring and replacement (21, 22) Pregnancy delayed for 12–18 months post-surgery (23)
Primary care and primary healthcare in obesity management	We recommend that PHPs identify PLWO, and initiate patient-centred, health-focused conversations with them. (Level 3; Grade C) We recommend that PHPs ensure that they ask PLWO for their permission prior to discussing weight or taking anthropometric measurements. (Level 3; Grade C) Primary care interventions should be used to increase health literacy in individuals’ knowledge about and skills in weight management as an effective intervention to manage weight. (Level 1a; Grade A) PHPs should refer PLWO to primary care multi-component programmes with personalised obesity management strategies as an effective way to support obesity management. (Level 1b; Grade B) PHPs can use collaborative deliberation with motivational interviewing to tailor action plans to individuals’ life context in a way that is manageable and sustainable to support improved physical and emotional health, and weight management. (Level 2b; Grade C) Interventions that target a specific ethnic group should consider the diversity of psychological and social practices with regard to excess weight, food and physical activity as well as socioeconomic circumstances, as they may differ across and within different ethnic groups. (Level 1b; Grade B) Longitudinal primary care interventions should focus on incremental, personalised, small behaviour changes (the ‘Small Changes’ approach) to be effective in supporting people to manage their weight. (Level 1b; Grade B) Primary care multi-component programmes should consider personalised obesity management strategies as an effective way to support PLWO. (Level 1b; Grade B) Primary care interventions that are behaviour based (nutrition, exercise, lifestyle), alone or in combination with pharmacotherapy, should be utilised to manage PLWO. (Level 1a; Grade A) Group-based nutrition and physical activity sessions informed by the Diabetes Prevention Program and the Look AHEAD (Action for Health in Diabetes) programme should be used as an effective management option for PLWO. (Level 1b; Grade A) Interventions that use technology to increase reach to larger numbers of people asynchronously should be a potentially viable lower-cost method in a community-based setting. (Level 1b; Grade B) Educators in undergraduate, graduate and continuing education programmes for PHPs should provide courses and clinical experiences to address the gaps in skills, knowledge of the evidence, and attitudes necessary to confidently and effectively support PLWO. (Level 1a; Grade A)	Non-stigmatised engagement Evidence for multi-component intervention combining: Nutrition Physical activity Small behaviour changes Sleep optimisation Aim is improved function and quality of live SA still lacks critical system level changes: Training of primary care providers Referral pathways
Commercial products and programmes in obesity management	We do not recommend the use of over-the-counter commercial weight loss products for obesity management, owing to lack of evidence. (Level 4; Grade D) We do not suggest that commercial weight loss programmes be used for improvement in blood pressure and lipid control in adults living with obesity. (Level 4; Grade D)	For adults living with overweight or obesity, some commercial programmes exist that should achieve mild-to-moderate weight loss over the short or medium term, and a mild reduction in glycated haemoglobin values over a short term in adults with type 2 diabetes compared with usual care or education HCP should be aware of local offerings and guide patients toward safe, evidence-based interventions
Emerging technologies and virtual medicine in obesity management	Implementation of strategies in the management of obesity can be delivered through web-based platforms (e.g. online education on medical nutrition therapy and physical activity) or mobile devices (e.g. daily weight reporting through a smartphone phone application). (Level 2a; Grade B) We suggest that healthcare providers incorporate individualised feedback and follow-up (e.g. personalised coaching or feedback via phone or email) into technology-based management strategies to improve weight loss outcomes. (Level 4; Grade D) The use of wearable activity-tracking technology should be part of a comprehensive strategy for weight loss. (Level 1a; Grade A)	International evidence for combining face-to-face care with technology such as: Mobile applications Telemedicine Wearable devices Web-based platforms Artificial intelligence (personalised care) Applicable to local context due to accessibility, cost-effectiveness, and scalability Local evidence lacking Important governance frameworks: Data privacy Data literacy Equitable access
Weight management over the reproductive years for adult women living with obesity	We recommend that HCPs should discuss weight management targets specific to the reproductive years with adult women living with obesity: pre-conception weight loss, gestational weight gain of 5 - 9 kg over the entire pregnancy, and postpartum weight loss of – at minimum – gestational weight gain to reduce the risk of adverse outcomes in the current or a future pregnancy. (Level 3 (preconception); Level 4 (gestational); Level 3 (postpartum); Grade C (preconception); Grade D (gestational); Grade C (postpartum)) HCPs should offer behaviour change interventions, including both nutrition and physical activity, to adult women living with obesity who are considering a pregnancy, who are pregnant, and who are postpartum in order to achieve weight targets. (Level 3 (preconception); Level 2a (gestational); Level 1a (postpartum); Grade C (preconception); Grade B (gestational); Grade A (postpartum)) We recommend that HCPs encourage and support pregnant women with obesity to consume foods consistent with a healthy dietary pattern in order to meet their target gestational weight gain. (Level 3; Grade C) We recommend that HCPs encourage and support pregnant women with obesity who do not have contraindications to exercise during pregnancy to engage in at least 150 minutes per week of moderate-intensity physical activity to assist in the management of gestational weight gain. (Level 3; Grade C) HCPs should not prescribe metformin for managing gestational weight gain in women with obesity (Level 1b, Grade A). We suggest no weight management medications during pregnancy or breastfeeding. (Level 1b (metformin); Level 4 (all medication); Grade A (metformin; Grade D (all medication)) We recommend that women with obesity be offered additional breastfeeding support owing to decreased rates of initiation and continuation. (Level 3; Grade C)	Monitor for gestational weight gain exceeding 5-9 kg for BMI ≥30 kg/m² (24) Evidence for pre-, ante-, and postnatal behavioural interventions (25) Breastfeeding offers health benefits (26) Avoid pharmacotherapy

HCP, health care practitioner; PLWO, people living with obesity; SA, South Africa; OT, occupational therapy; BMI, body mass index; ADHD, Attention Deficit Hyperactivity Disorder; MNT, medical nutrition therapy; BP, blood pressure; T2DM, type 2 diabetes mellitis; OSA, obstructive sleep apnea; MBS, metabolic and bariatric surgery); QoL, quality of life; PA, physical activity; MBS, metabolic and bariatric surgery; MASLD, metabolic associated steatotic liver disease; MDT, multidisciplinary team; PHP, primary healthcare provider.

#### Epidemiology of adult obesity

In South Africa (SA), between 1998 and 2017, adult obesity prevalence rose by 38% to 28.0% with the rate of increase being double in women (29.5%→42.6%) compared to men (10.1% →12.1%) (27). Similar trends were observed for central/abdominal obesity, with higher rates of increase in women than men (60%→77.2%; 21.6%→26.3% respectively) and higher rates of increase observed in rural SA provinces. Epidemiological data in SA is dated with the last national survey conducted in 2016/2017 (28).

Obesity is associated with increased risk of diseases such as cardiovascular disease, diabetes and cancer, and accounted for 11.1% of total deaths and 6.9% of disability adjusted life years in 2012 (29–31). Public-sector treatment costs were estimated at ZAR33.194 million in 2020 (0.67% of gross domestic product) and private insurer cost at ZAR21.8 billion in 2022 (32). Given increasing obesity trajectories and large SA data gaps, regular surveillance is required to reduce the unequal burden and escalating costs.

Recommendations were adopted from the Canadian CPG (**Table 1**).

#### The science of obesity

Obesity is a complex chronic disease in which abnormal or excess body fat (adiposity) impairs health, increases the risk of long-term medical complications, and reduces lifespan (33). A complex interaction of genetic, metabolic, behavioral, and environmental factors culminate in dysfunction of energy balance (34). The brain regulates energy and appetite in the hypothalamus (homeostatic control), mesolimbic system (hedonic drive), and frontal lobe (cognitive functioning) with all regions integrating signals from the gut and adipose tissue (35, 36).

Obesity may be classified as genetic, secondary, or primary obesity (**Figure 1**), the latter with non-modifiable causes and potentially modifiable contributors. Two interrelated concepts underpin obesity pathophysiology: the global rise in body weight and the personal fat threshold (37). Genetic susceptibility, combined with a modern toxic environment and poor diet quality disrupts energy balance, driving population weight gain and pushing individuals beyond their personal fat threshold (38). Visceral fat is metabolically distinct from subcutaneous adipose tissue, exhibiting greater insulin resistance, pro-inflammatory activity, and stronger associations with cardiometabolic disease (39).

**Figure 1:**
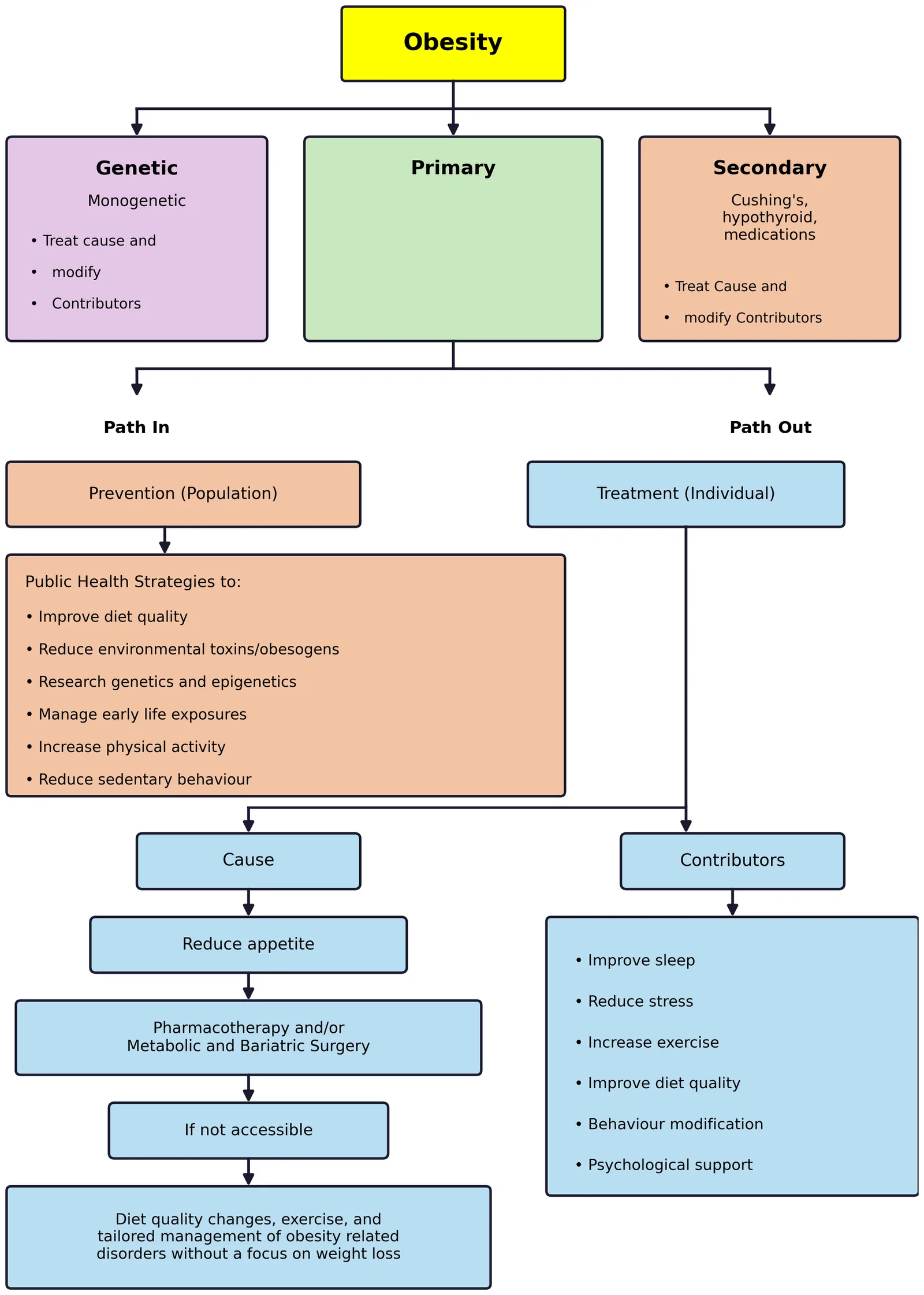
Rational approach to obesity management.

#### Prevention and harm reduction of obesity (clinical prevention)

Obesity prevention requires action across primary, secondary and tertiary levels, recognizing causes and contributors beyond diet and exercise including medications and social and environmental determinants (40).

Primary prevention evidence is strongest in pregnancy where interventions lower the risk of excessive gestational weight gain, particularly when delivered within prenatal care (41). Secondary prevention emphasizes early detection and prevention of further weight gain in early stages while, tertiary prevention aligns with weight-loss management for established comorbidity.

While smoking cessation results in weight gain, cardiovascular and mortality benefits outweigh the risk, and short-term strategies may limit weight gain (42). Medication-associated weight gain (antipsychotics, antidepressants, antihyperglycaemics, corticosteroids and antiretrovirals) warrants choosing weight-neutral agents or instituting monitoring and behavioral support (43). Menopause and young adulthood are high-risk stages for weight gain. Behavioral programs can attenuate gain in midlife women (44) while effects in young adults are modest and not sustained (45).

#### Enabling participation in activities of daily living for people living with obesity

PLWO frequently face barriers to self-care, mobility, and healthcare participation (46). Fall risk assessment using the Falls Efficacy Scale is recommended across obesity classes (13). and routine skin integrity screening with practical moisture and friction reducing strategies should be instituted (47). Timeous referral to occupational therapy, physiotherapy and biokinetics supports independence, equipment selection, and physical activity counselling – though local SA evidence and access remain limited (48).

Recommendations were adopted from the Canadian CPG (**Table 1**).

#### Assessment of people living with obesity

Goals of assessment in PLWO are to confirm the diagnosis, determine disease severity, identify comorbidities and contributors to weight, and guide management in a stigma-free environment. Healthcare providers (HCPs) should initiate discussions with empathy, using frameworks such as the 5As (Ask, Assess, Advise, Agree, Assist) to explore readiness, set goals, and support long-term behavior change (14).

Screening relies on anthropometric measures and while body mass index (BMI) remains widely used it cannot distinguish fat from muscle or indicate fat distribution. Combining BMI with waist circumference or waist-to-height ratio better defines cardiometabolic risk (33). Ethnic-specific cut-offs are critical as evidenced by increased cardiometabolic risk in Asian populations at lower BMI thresholds (49). For South Africans, WHO thresholds apply, even though emerging evidence suggests different waist circumference cut-points for black populations (50).

Assessment includes an obesity-centered history (medical, weight trajectory, nutrition, physical activity, mental health, sleep, substance use, and social context) and a focused physical examination (anthropometry, cardiovascular, musculoskeletal, endocrine systems and skin). Laboratory investigations should screen for diabetes, dyslipidaemia, steatotic liver disease - including the FIB-4 score (51), and obstructive sleep apnea (OSA), guided by clinical presentation.

Tools such as the Edmonton Obesity Staging System (EOSS) provide a multidimensional view of disease impact beyond BMI, predicting morbidity and mortality (17). The 4Ms framework (Mental, Mechanical, Metabolic, Monetary) further helps identify drivers, complications, and barriers (52). Patient-reported outcome measures (PROMs) are increasingly recommended to capture quality-of-life domains (53).

Recommendations were adapted from the Canadian CPG, with updates in terminology only (**Table 1**).

#### The role of mental health in obesity management

Individuals with mental illness face higher risks of obesity due to biological, psychological, and social factors, compounded by weight-promoting psychotropic medications (54). Conversely, obesity increases vulnerability to depression, anxiety, binge-eating disorder (BED), and attention deficit hyperactivity disorder (ADHD) (54, 55).

Screening for mental illness in PLWO is essential, particularly before MBS, as psychiatric comorbidities can influence postoperative outcomes and adherence (56). Antipsychotic-induced weight gain is a significant clinical issue in those with mental illness and behavioral and lifestyle interventions show modest benefit (57), while pharmacological options may mitigate risk (58).

Recommendations were adopted from the Canadian CPG (**Table 1**).

#### Medical nutrition therapy in obesity management

Medical nutrition therapy (MNT) focuses on improving health outcomes rather than solely achieving weight loss, fostering a positive relationship with food. Interventions should be personalized, culturally appropriate, affordable, and sustainable, and ideally delivered by registered dietitians if available.

There is no single best dietary approach and success depends on long-term adherence (59). Options include calorie-restricted plans, macronutrient-based approaches, and patterns such as Mediterranean, vegetarian, Nordic, and Portfolio diets which may improve glycaemic, blood pressure, and lipid profiles (60). Food-based strategies - emphasizing pulses, fruits, vegetables, nuts, whole grains, and dairy—also show benefits (61). Meal replacements and intermittent fasting may support short-term weight loss but require medical oversight especially in older adults or those at risk of malnutrition (62, 63).

Caloric restriction can achieve initial weight loss but is difficult to sustain due to physiological adaptations that increase hunger and promote weight regain (64). Non-restrictive approaches, including mindful eating and Health at Every Size^®^ (HAES^®^) principles aim to reduce weight stigma and improve psychological wellbeing, though evidence remains limited (65). Behavioral support is essential, with intensive lifestyle programs demonstrating significant improvements in overall health (66).

Recommendations were adapted from the Canadian CPG, improving concepts and terminology only (**Table 1**).

#### Physical activity in obesity management

The World Health Organization (WHO) defines physical activity as bodily movement by skeletal muscles that requires energy expenditure (67). Physical inactivity remains one of the leading modifiable risk factors for mortality (68).

While exercise alone produces modest weight reduction (2–3 kg), its value lies in improving health, mobility, and muscle mass. It reduces abdominal visceral fat and ectopic fat, even without significant weight change, and improves insulin sensitivity, blood pressure, and lipid profiles (69).

Aerobic and resistance training enhance cardiorespiratory fitness, strength, and muscle mass preservation, which is critical during weight loss interventions. High-intensity interval training (HIIT) may improve fitness and metabolic markers in less time, though its superiority for fat loss remains unclear, and behavioral support is essential for sustained change (66, 70).

South African guidance align with the WHO Global Action Plan on Physical Activity (71) and the National Strategy for Obesity 2023–2028 (6), which aims to reduce physical inactivity by 15% by 2030.

Recommendations were adopted from the Canadian CPG (**Table 1**).

#### Effective psychological and behavioral interventions in obesity management

Psychological and behavioral interventions are essential for effective obesity management, as sustained lifestyle change is key to improving health and quality of life. These interventions aim to support PLWO in adopting and maintaining healthier eating patterns, physical activity, and treatment adherence. MNT and physical activity interventions rarely succeed without addressing psychological drivers of behavior (66).

Behavioral interventions are most effective when they are person-centered, responsive to the expressed needs of PLWO and combine behavior modification (goal setting, self-monitoring, problem solving), cognitive strategies (reframing unhelpful beliefs, enhancing self-efficacy), and value-based approaches that align with personal priorities (72). Interventions should be culturally adapted and build executive control skills and resilience - planning, impulse regulation, and problem solving - while managing weight-loss expectations to prevent disengagement (73). Acceptance and Commitment therapy and compassion-focused strategies help address internalized weight bias and stigma, which often undermine motivation and outcomes. Digital tools may augment interventions, though uptake remains a challenge (74).

Recommendations were adopted from the Canadian CPG (**Table 1**).

#### Pharmacotherapy for obesity management

Pharmacological agents used in treatment of adults with a body mass index (BMI) ≥30 kg/m² or ≥27 kg/m² with adiposity-related complications is not a stand-alone solution but part of holistic obesity management alongside MNT, physical activity, psychological support and MBS (75, 76).

In South Africa, five drugs are approved for chronic obesity management: orlistat, liraglutide, naltrexone/bupropion, semaglutide and tirzepatide (**Table 2**). Regulatory approval of medication has changed significantly since publication of the guideline and updates are being drafted. Agents differ in mechanism, efficacy, tolerability, and cost. Phentermine is registered for short-term use only. Emerging therapies such as cagrilintide (amylin analogue), and dual and triple agonists show promise. The use of off-label or unapproved therapies is strongly discouraged.

**Table 2:** Pharmacotherapy for obesity in adults in South Africa.

	Orlistat	Naltrexone/Bupropion	Liraglutide	Semaglutide	Tirzepatide
Mode of administration	Oral	Oral	Subcutaneous	Subcutaneous	Subcutaneous
Dose/frequency	120 mg TID	16/180 mg bd (8/90 mg per tablet)	3 mg daily	2.4 mg weekly	5, 10, 15 mg weekly
Titration schedule	No titration	1 tablet daily x 1 week then 1 tablet BID daily x 1 week then 2 tablets in AM, 1 tablet in PM x 1 week then 2tablets BID	0.6 mg daily x 1 week then 1.2 mg daily x 1 week then 1.8 mg daily x 1 week Then 2.4 mg daily x 1 week Then 3 mg daily	0.25 mg weekly x 1 month then 0.5 mg weekly x 1 month then 1 mg weekly x 1 month then 1.7 mg weekly x 1 month then 2.4 mg weekly s.c	2.5 mg weekly x 1 month then 5 mg weekly x 1 month If increase indicated after 4 weeks at 5 mg, then 7.5 mg weekly x 1 month then 10 mg weekly x 1 month If increase indicated after 4 weeks at 10 mg, then 12.5 mg weekly x 1 month then 15 mg weekly
% weight loss, placebo subtracted	2.9% at 1 year (95% CI −3.35 to −2.50)	4.8% at 56 weeks (95% CI 4.0-5.6)	5.6kg at 56 weeks (95% CI 5.1-6.0)	12.4% at 68 weeks (95% CI 11.5–13.4)	11.9% (5 mg) (95% CI 10.4–13.4) 16.4% (10 mg) (95% CI 14.8–17.9) 17.8% (15 mg) (95% CI 16.3–19.3) @ 72 weeks
Effect on weight over longer term, placebo subtracted	2.8 kg at 4 years (p<0.001)	Not studied	4.3% at 3 years (95% CI 3.7-4.9)	12.6% at 104 weeks (95% CI 9.8–15.3)	11.1% (5mg) (95% CI 7.8-14.4) 17.5% (10mg) (95% CI 11.3-23.6) 18.4% (15 mg) (95% CI 14.7-22.2) @176 weeks
% of patients achieving ≥ 5 % weight loss	54% (vs 33% with placebo)	48%(vs 16% with placebo)	63.2% (vs 27.1% with placebo)	86.4% (vs 31.5% with placebo)	85.1% (5mg) (95% CI 81.6-88.6) 88.9 % (10 mg) (95% CI 85.9-91.9) 90.9 % (15 mg) (95% CI 88.0–93.8) 34.5% placebo (95% CI 29.8–39.2)
% of patients achieving ≥ 10 % weight loss	26% (vs 14% with placebo)	25% (vs 7 % with placebo)	33.1% (vs 10.6% with placebo)	69.1% (vs 12% with placebo)	68.5 %(5mg) (95% CI 64.5–72.5) 78.1 % (10mg) (95% CI 74.4–81.7) 83.5 % (15 mg) (95% CI 80.0–86.9) 18.8 placebo (95% CI 14.9–22.7)
% of patients achieving ≥ 15% weight loss	Not studied	12% (vs 2% with placebo)	14.4% (vs 3.5% with placebo)	50.5% (vs 4.9% with placebo)	48 % (5mg) (95% CI 43.9–52.1) 66.6 % (10 mg) (95% CI 62.6–70.6) 70.6% (15mg) (95% CI 66.7–74.5) 8.8 % placebo (95% CI 5.9–11.7)
% of patients achieving ≥ 20 % weight loss	Not studied	Not studied	Not studied	Statistical significance not tested	30% (5mg) (95% CI 26.4–33.6) 50.1 % (10mg) (95% CI 46.0–54.2) 56.7% (15 mg) (95% CI 52.6–60.8) 3.1 % placebo (95% CI 1.1–5.1)
Effect on maintenance of previous lifestyle-induced weight loss	2.4 kg less weight regain vs placebo over 3 years	Not studied	6.2% additional placebo-subtracted weight loss at 1 year	Not studied	94% of patients able to maintain at least 80 % of weight lost vs placebo 43.8% (OR 19.7, 95% CI 10.3-37.6, p<0.001)
Contraindication	Cholestasis Chronic malabsorption syndrome Pregnancy, women attempting conception, breastfeeding	Uncontrolled hypertension Any opioid use History of, or risk factors for, seizure Abrupt discontinuation of alcohol Concomitant administration of monoamine oxidase inhibitors (MAOI) Severe hepatic impairment End-stage renal failure Pregnancy, women attempting conception, breastfeeding	Personal or family history of medullary thyroid cancer Personal history of MEN2 syndrome Pregnancy, women attempting conception, breastfeeding	Personal or family history of medullary thyroid cancer Personal history of MEN2 syndrome Pregnancy, women attempting conception, breastfeeding	Personal or family history of medullary thyroid cancer Personal history of MEN2 syndrome Pregnancy, women attempting conception, breastfeeding
Common side effects	Loose, oily stools, flatus	Nausea, constipation, headache, dry mouth, dizziness, diarrhoea	Nausea, constipation, diarrhoea, vomiting	Nausea, constipation, diarrhoea, vomiting	Nausea, constipation, diarrhoea, dyspepsia, vomiting
Rare side effects	Liver failure Nephrolithiasis Acute kidney injury	Seizure Worsening of depression	Cholelithiasis Pancreatitis	Cholelithiasis Pancreatitis	Cholelithiasis Pancreatitis
Drug interactions	May reduce availability of oral contraceptives Fat-soluble vitamins Levothyroxine Cyclosporine Oral anticoagulants Anticonvulsants	Yes	Reduces absorption of oral contraceptive during titration phase; switch to a non-oral contraceptive method or add a barrier method of contraception for 4 weeks after initiation and for 4 weeks after each dose escalation	Reduces absorption of oral contraceptive during titration phase; switch to a non-oral contraceptive method or add a barrier method of contraception for 4 weeks after initiation and for 4 weeks after each dose escalation	Reduces absorption of oral contraceptive during titration phase; switch to a non-oral contraceptive method or add a barrier method of contraception for 4 weeks after initiation and for 4 weeks after each dose escalation
Cost	$$	$$$	$$$ (clone available)	$$$$	$$$$

CI, Confidence Interval.

Pharmacotherapy benefits extend to comorbidities such as cardiovascular disease, T2DM, OSA, steatotic liver disease, and osteoarthritis (77, 78). However, response is heterogeneous and discontinuation leads to weight regain, underscoring the need for long-term use. Recommendations were adopted from the Canadian CPG (**Table 1**) though this was subsequently updated (75).

#### Metabolic and bariatric surgery: selection and preoperative work-up

Surgical treatment is the most robust long-term treatment for obesity, outperforming behavioral and medical interventions (18, 20). Indications include a BMI >35 kg/m² regardless of comorbidities, and BMI 30–34.9 kg/m² with metabolic disease (19).

Preoperative assessment include screening for micronutrient deficiencies (especially vitamin D and iron), OSA, cardiac and pulmonary risk, and mental health status (15, 22, 79). Smoking cessation is encouraged to reduce postoperative complications (80). A low-calorie diet for 2–3 weeks before surgery helps reduce liver size and operative difficulty and medication adjustment especially for T2DM and anticoagulation during this time is critical (81). Recommendations were adopted from the Canadian CPG (**Table 1**).

#### Metabolic and bariatric surgery: surgical options and outcomes

MBS achieves 20–40% total weight loss and high remission rates for T2DM, hypertension, dyslipidaemia, OSA, and steatotic liver disease (19). It improves quality of life, reduces cancer risk, and lowers long-term mortality by up to 49%, with median life expectancy gains of 6–9 years (19). Published outcomes after MBS from South African series are limited but consistent with international reports (82, 83). Although MBS was historically only accessible through private health insurance, public centers now have established MBS programs (84).

The most common procedures performed are sleeve gastrectomy and Roux-en-Y gastric bypass. Sleeve gastrectomy is technically simpler with a 30% risk of developing gastroesophageal reflux postoperatively, while RYGB carries a risk of internal herniation and stomal ulceration but remains the gold standard for sustained metabolic benefits (85). Endoscopic sleeve gastroplasty is now recognized as a less invasive option in the treatment armamentarium (86). Emerging options are the single-anastomosis duodenal-ileal bypass and one-anastomosis gastric bypass, performed only when patient outcomes are captured in a national registry (87, 88).

MBS should be planned following multidisciplinary discussion, considering goals, comorbidities, and perioperative and long-term risks. Perioperative care is provided by a trained and accredited team following enhanced recovery after surgery protocols (89). Recommendations were adapted from the Canadian CPG, as indications were updated (**Table 1**).

#### Metabolic and bariatric surgery: postoperative management

Postoperative care is shared between the MBS multidisciplinary team, primary healthcare providers, and PLWO. Priorities include lifelong adherence to nutrition plans, adequate protein intake, vitamin and mineral supplementation, and regular physical activity (90). Pregnancy should be delayed for 12–18 months after surgery (23).

Annual micronutrient monitoring and replacement is well defined to limit deficiencies in iron, vitamin B12, vitamin D, calcium, and fat-soluble vitamins (22). Long-term complications include dumping syndrome, bone loss, and nephrolithiasis. Psychological support aims to diagnose and treat depression, body image issues and addiction. Weight regain occurs in up to 35% of patients and should be managed through MNT reinforcement, psychological support, and, if necessary, pharmacotherapy or surgical revision (91). Recommendations were adopted from the Canadian CPG (**Table 1**).

#### Primary care and primary healthcare in obesity management

Primary healthcare providers play a pivotal role as first contact for PLWO. Effective care demands respectful, patient-centered conversations that avoid stigma and use structured frameworks, collaborative deliberation, and motivational interviewing (14). These approaches prioritize health-focused goals, such as improved function and quality of life, over weight alone.

Multi-component interventions combining MNT, physical activity and behavioral strategies outperform single-component approaches (92). Cultural tailoring and health literacy are essential for engagement, particularly in diverse and resource-constrained settings (93). System-level changes - training of primary health care providers, creating referral pathways, and leveraging technology-enabled models - are critical for scalability and sustainability. Recommendations were adopted from the Canadian CPG (**Table 1**).

#### Commercial products and programs in obesity management

The weight loss industry is vast and often characterized by unrealistic claims. While some commercial programs may achieve modest short-term weight loss and marginal glycemic improvements, evidence for sustained benefits - or impact on blood pressure and lipids - is lacking (94). Over-the-counter supplements are widely available but unproven in high-quality trials and may pose health risks (95). Healthcare providers should familiarize themselves with local offerings and guide patients toward safe, evidence-based options, while PLWO should critically evaluate commercial claims. Recommendations were adapted from the Canadian CPG and specific products and programs were not mentioned (**Table 1**).

#### Emerging technologies and virtual medicine in obesity management

Emerging technologies in obesity management encompass digital health tools such as telemedicine, mobile apps, wearable devices, web-based platforms, and artificial intelligence (AI). Innovations enhance accessibility, convenience, and cost-effectiveness while maintaining supportive elements of conventional care. Interventions include self-monitoring, goal setting, and personalized feedback delivered via smartphones, text messaging, or wearable trackers.

Evidence suggests these strategies can improve short-term weight loss, particularly when combined with face-to-face care, but long-term data remain limited (96). Hybrid models integrating digital and in-person approaches show promising results for weight reduction and physical activity and may be especially applicable to SA. Challenges include short study durations, methodological limitations, and underrepresentation of men and diverse populations. Ethical considerations such as data privacy (POPIA compliance), digital literacy, and equitable access are critical, especially in resource-limited settings (97). Recommendations were adopted from the Canadian CPG (**Table 1**).

#### Weight management over the reproductive years for adult women living with obesity

Weight management during the reproductive years is critical for women with obesity due to increased risks of infertility, gestational diabetes, hypertensive disorders, caesarean delivery, and adverse neonatal outcomes (98, 99). During pregnancy, achieving guideline-concordant gestational weight gain of 5–9 kg for BMI ≥30 kg/m² is essential, as excess gain is linked to complications such as macrosomia, preterm birth, and postpartum weight retention (24). Evidence supports behavioral interventions as the most effective strategies, while pharmacotherapy is generally avoided (100). Postpartum weight retention is common and contributes to long-term obesity and complications in future pregnancies, while breastfeeding offers health benefits but may require additional support for women with obesity (26). Recommendations were adopted from the Canadian CPG (**Table 1**).

### Model of care, referral & health-system integration

Management of obesity follows disease classification - genetic, secondary and primary (**Figure 1**). For primary obesity, the “path in” is driven by environmental factors such as sleep habits, stress, medications and early-life programming. This differs from the “path out”, which targets the final common pathway of increased appetite. Effective treatment therefore prioritizes appetite-modulating therapies including GLP-1 receptor agonists and MBS - supported by MNT, physical activity and behavioral and psychological care (34). Prevention and harm-reduction strategies operate at population and primary health care levels. Early detection and non-stigmatizing care should be honored throughout.

## Research gaps and future directions

### Culture and ethnicity

Limited local evidence suggests a tendency to underestimate body weight and highlights varied perceptions of body image, body size, healthy food choices, obesity related health, and the Health Promotions Levy (Sugar Tax) (101–103).

While cultural beliefs, traditional body ideals, and community norms profoundly influence obesity perceptions and treatment-seeking behaviors, literature does not adequately represent SA’s diverse ethnic groups, languages, and socioeconomic contexts. Rather than providing guidance based predominantly on high-income country research or extrapolating inappropriately from other settings, we explicitly acknowledge this evidence gap and prioritize it as a critical research area for future guideline iterations.

### Obesity in people living with HIV

This guideline is placed in a context of high HIV prevalence, with nearly 6 million South Africans receiving lifelong antiretroviral therapy (ART) (104). However, evidence linking HIV infection, ART exposure, and obesity-related outcomes remains insufficient to support local guidance. People living with HIV (PWH) may have an increased risk of cardiovascular and metabolic disease, including myocardial infarction, stroke, and T2DM, which now account for the growing share of premature deaths (105–108). Prevention trials such as REPRIEVE, demonstrate targeted interventions can reduce major adverse cardiovascular events, even among PWH traditionally classified as low risk, but these alone are unlikely to offset cardiometabolic risk in the setting of prevalent and accelerating obesity (109).

Dolutegravir-based ART used by most PWH in South Africa is central to SA’s HIV program and is unlikely to be displaced in the near future (110, 111). Across African and international cohorts, dolutegravir-associated weight gain is often rapid and clinically meaningful, with disproportionate effects among women and black PWH (112, 113). SA trial data further show weight gain is accompanied by rising blood pressure, incident hypertension, and increased cardio–kidney-metabolic risk (114).

Despite these observations, three evidence gaps limit guideline development. First, the biological meaning of post-ART weight gain in PWH remains unclear. Unlike in the general population, weight gain in PWH may reflect preferential expansion of visceral and ectopic fat rather than generalized adiposity, driven by chronic inflammation, prior treatment effects, and reproductive life stages (including pregnancy and menopause) (115–117). Without SA-specific longitudinal data, clinicians cannot reliably distinguish metabolic harm from immune recovery. These uncertainties are compounded by overlapping HIV and weight stigma, where weight gain may be interpreted as wellness and weight loss as illness, delaying risk recognition and complicating counselling (118, 119).

Second, evidence is limited on when obesity-related complications emerge in PWH, by ART regimen and baseline adiposity. Third, although pharmacotherapy is well studied in the general population, their real-world integration into HIV care remains an evidence gap, particularly regarding safety, acceptability, retention, and delivery within in public-sector services (120, 121). Progress requires SA-based prospective cohorts and pragmatic studies within HIV care platforms. Without this evidence, guidance is based on imported thresholds rather than context-appropriate screening, staging, and treatment pathways for obesity in PWH.

## Conclusion and recommendations

This guideline adaptation represents a landmark in South Africa’s obesity response, providing clinicians with the country’s first national evidence-based guideline for adult obesity management. The committee produced a rigorous, locally relevant guideline efficiently and cost-effectively, underpinned by a transparent GRADE-ADAPTE methodology and multidisciplinary stakeholder engagement.

Challenges remain, including the absence of routine obesity surveillance, resource constraints, and limited local research funding. Successful implementation will require integration with national NCD strategies, workforce training, community outreach, and ongoing monitoring and evaluation.
